# Integrated transcriptomic and epigenetic data analysis identifies aberrant expression of genes in acute myeloid leukemia with MLL-AF9 translocation

**DOI:** 10.3892/mmr.2021.12295

**Published:** 2021-07-15

**Authors:** Fangce Wang, Zheng Li, Guangming Wang, Xiaoxue Tian, Jie Zhou, Wenlei Yu, Zhuoyi Fan, Lin Dong, Jinyuan Lu, Jun Xu, Wenjun Zhang, Aibin Liang

Mol Med Rep 21: 883-893, 2020, DOI: 10.3892/mmr.2019.10849

Following the publication of this paper, the authors have realized that the final article did not indicate in the Authors’ Contribution section that Fangce Wang and Zheng Li made equal contributions to this work (FW and ZL performed most of the statistical analyses and drafted the initial version of the manuscript). Therefore, the affiliations for this paper should have been written as follows (changes are highlighted in bold):

FANGCE WANG^1*^, ZHENG LI^1*^, GUANGMING WANG^1^, XIAOXUE TIAN^1^, JIE ZHOU^1^, WENLEI YU^1^, ZHUOYI FAN^1^, LIN DONG^1^, JINYUAN LU^1^, JUN XU^2^, WENJUN ZHANG^1^ and AIBIN LIANG^1^

^1^Department of Hematology, Tongji Hospital, Tongji University School of Medicine, Shanghai 200092; ^2^Medical Center for Stem Cell Engineering and Transformation, East Hospital, Tongji University School of Medicine, Shanghai 200120, P.R. China

*Contributed equally

The authors confirm that there are no further errors in the paper, and all the authors agree to this correction. The authors and the Editor apologize for any inconvenience caused.

